# Dietary protein levels modulate the gut microbiome composition through fecal samples derived from lactating ewes

**DOI:** 10.3389/fendo.2023.1194425

**Published:** 2023-08-09

**Authors:** Jiachong Liang, Sikandar Ali, Chunrong Lv, Hongyuan Yang, Xiaoqi Zhao, Xiaojun Ni, Chunyan Li, Baiji Danzeng, Yajing Wang, Guobo Quan

**Affiliations:** ^1^ The Small Ruminant Department, Yunnan Animal Science and Veterinary Institute, Kunming, Yunnan, China; ^2^ Zhejiang Vegamax Biotechnology Co., Ltd, Hangzhou, Zhejiang, China; ^3^ State Key Laboratory of Animal Nutrition, Beijing Engineering Technology Research Center of Raw Milk Quality and Safety Control, College of Animal Science and Technology, China Agricultural University, Beijing, China

**Keywords:** sheep, lactation, dietary protein, gut microbiome, fecal microbiome, metagenomics

## Abstract

In ruminants, the digestion and utilization of dietary proteins are closely linked to the bacterial populations that are present in the gastrointestinal tract. In the present study, 16S rDNA sequencing, together with a metagenomic strategy was used to characterize the fecal bacteria of ewes in the early lactation stage after feeding with three levels of dietary proteins 8.58%, 10.34%, and 13.93%, in three different groups (H_1), (H_m) and (H_h), respectively. A total of 376,278,516 clean data-points were obtained by metagenomic sequencing. Firmicutes and Bacteroidetes were the dominant phyla, regardless of the dietary protein levels. In the H_h group, the phyla Proteobacteria, Caldiserica, and Candidatus_Cryosericota were less abundant than those in the H_I group. In contrast, Lentisphaerae, Chlamydiae, and Planctomycetes were significantly more abundant in the H_h group. Some genera, such as Prevotella, Roseburia, and Firmicutes_unclassified, were less abundant in the H_h group than those in the H_I group. In contrast, *Ruminococcus, Ruminococcaceae_noname, Anaerotruncus, Thermotalae, Lentisphaerae_noname*, and *Paraprevotella* were enriched in the H_h group. The acquired microbial genes were mainly clustered into biological processes; molecular functions; cytosol; cellular components; cytoplasm; structural constituents of ribosomes; plasma membranes; translation; and catalytic activities. 205987 genes were significantly enriched in the H_h group. In contrast, 108129 genes were more abundant in the H_I group. Our findings reveal that dynamic changes in fecal bacteria and their genes are strongly influenced by the levels of dietary proteins. We discovered that differentially expressed genes mainly regulate metabolic activity and KEGG demonstrated the primary involvement of these genes in the metabolism of carbohydrates, amino acids, nucleotides, and vitamins. Additionally, genes responsible for metabolism were more abundant in the H_h group. Investigating fecal bacterial characteristics may help researchers develop a dietary formula for lactating ewes to optimize the growth and health of ewes and lambs.

## 1 Introduction

Breeders and researchers are exploring better ways to improve the productive traits (milk, meat and wool production) of sheep breeds through different strategies, but the sheep breed is a critical factor influencing productive traits (1). However, daily diets, including their sources and components, also affect sheep growth and health (1, 2). Generally, sheep graze on pastures growing different types of plants such as grasses, clovers, and weeds. During certain harsh periods of the year, sheep moved to temporary indoor rearing and were mostly fed with small amounts of concentrates and hay (1, 3). Currently, the feeding system of sheep is changing significantly and gradually moving to an indoor-rearing mode (2). Therefore, determining the effects of nutrition on sheep-production during indoor rearing has become critical (2, 3).

Proteins are important nutrients for ruminants and play important roles in metabolic activities (4). Many tissues and organs are involved in the digestion of dietary proteins (5). Generally, a large proportion of dietary proteins is degraded by ruminal bacteria to small peptides and NH_3_, which are further utilized by bacteria to synthesize bacterially derived proteins (5). These proteins are further digested into amino acids and small peptides and are absorbed by the small intestine (5, 6). These digested products enter the mesenteric and portal veins. After they enter the liver, through synthesizing and degrading activities, these components are utilized through the surrounding tissues and organs (4, 6). The use of dietary proteins is closely associated with gastrointestinal bacterial communities. The composition and diversity of gastrointestinal bacteria greatly influence the health status (7) and productivity of host animals (8, 9).

Ruminants depend on a diverse and complex population of microbial organisms in their gastrointestinal tracts to convert their food into usable forms of nutrients (4). In ruminants, the gut, particularly the rumen, acts as the primary fermentation chamber for the microbial community (7, 8). Symbiotic bacterial communities are crucial for host production and health in many ways, such as balancing immune responses, utilizing nutrients, and regulating physiological activities (7). In sheep, a previous study analyzed the composition and diversity of bacteria in the gastrointestinal tract, including the rumen, reticulum, omasum, abomasum, duodenum, jejunum, ileum, cecum, colon, and rectum (10). However, problems related to animal use in research exist, particularly when collecting rumen fluid from ruminants (11, 12). In addition, studies have shown a potential relationship between fecal bacterial communities and rumen bacteria.

Fecal samples are usually collected and used to assess the ruminant gastrointestinal microbiota (13). According to a report by Shanks et al., fecal bacteria play crucial roles in animal health and productivity, as well as in food safety, pathogenicity, and also in methods of fecal-pollution-detection (14). Several studies on fecal bacteria have been conducted in sheep (10, 13) and cattle (14, 15). Although previous investigations have confirmed the functional roles of dietary proteins in the growth and development of sheep (2, 3, 5), there are no reports on the impact of dietary proteins on fecal bacteria in the first 90 days of lactating after the parturition. In this study, we aimed to characterize the fecal bacterial community of *Yunnan semi-fine wool sheep* at the lactating stage, using 16S rDNA sequencing together with metagenomic approaches, based on growth data obtained after feeding with various levels of dietary proteins. Investigating fecal bacterial diversity in lactating ewes may be useful for developing a dietary formulation for optimizing the growth and health of ewes and lambs.

## 2 Materials and methods

### 2.1 Ethics statement

In the present study, all experiments trials were approved by the ethical committee of the Yunnan Animal Science and Veterinary Institute (201911004). Moreover, all authors strictly followed the approved protocols and guidelines by the State Science and Technology Commission of the People’s Republic of China, 1988 and the Standing Committee of Yunnan Provincial People’s Congress 2007.10).

### 2.2 Collection of fecal samples

The feeding experiment was conducted at the Yunnan Animal Science and Veterinary Institute farm, Kunming City, People’s Republic of China (26°22′N; 103°40′E). Eighteen ewes with similar body weight (approximately 50 kg) and age (2 years) were used in this study. The details of the feed with different dietary protein levels and formulations are presented in **Table 1**, which were slightly modified based on a previous study (16). According to the levels of proteins used (8.58%, 10.34%, and 13.93%), 18 ewes were equally distributed into three groups H_I group, H_m group, and H_h groups. Each ewe was housed separately and provided with dietary feed from the 135^th^ day of pregnancy till 90^th^ day after parturition. All ewes were fed twice daily and had access to water at libitum. Fresh fecal samples were collected from the terminal rectum ninety days after parturition to avoid contamination (17). Approximately 10 g of fecal sample was loaded into a 10 mL sterile freezing tube (BIOFIL, China) and immediately frozen in a liquid nitrogen tank. Finally, the tubes were stored at –80°C for the subsequent experiments.

**Table 1:** The diet components and nutritional levels of ewes (air-dry basis).

Corn raw material	H_I	H_m	H_h
corn	28.2	26	19.05
soybean meal	5.40	8.65	18.60
corn starch	8.65	7.60	4.70
calcium carbonate	0.55	0.60	0.65
calcium hydrogen phosphate	0.55	0.50	0.35
salt	0.30	0.30	0.30
baking soda	0.35	0.35	0.35
premix	1.00	1.00	1.00
corn silage	40.00	35.00	34.00
bean powder	2.00	10.00	11.00
wheat straw	13.00	10.00	10.00
total	100	100	100
fine to coarse ratio	45:55	45:55	45:55
Nutrient content			
metabolizable energy	9.45	9.47	9.47
protein	8.58	10.34	13.93
neutral detergent fiber (NDF)	32.17	32.01	32.52
acid detergent fiber (ADF)	17.24	17.71	18.44
calcium	0.69	0.71	0.71
phosphorus	0.38	0.39	0.39

### 2.3 DNA extraction

Total DNA was extracted from fecal samples using the E.Z.N.A. ^®^Stool DNA Kit (D4015, Omega, Inc., USA) in accordance with the manufacturer’s instructions. Nuclear-free water was used as the blank. The total DNA was eluted using 50 μL of the Elution buffer, and finally stored at -80°C.

### 2.4 PCR amplification and 16S rDNA sequencing

The V3-V4 region of the prokaryotic (bacterial and archaeal) small-subunit (16S) rRNA gene was amplified using primers 341F (5’-CCTACGGGNGGCWGCAG-3’) and 805R (5’-GACTACHVGGGTATCTAATCC-3’). The 5’ ends of the primers were tagged with specific barcodes per sample and sequenced using universal primers. PCR amplification was performed in a total volume of 25 μL reaction mixture containing 25 ng of template DNA, 12.5 μL PCR Premix, 2.5 μL of each primer, and PCR-grade water to adjust the total volume. The PCR settings to amplify the prokaryotic 16S fragments consisted of an initial denaturation at 98°C for 30 s, 32 cycles of denaturation at 98°C for 10 s, annealing at 54°C for 30 s, extension at 72°C for 45 s, and then final extension at 72°C for 10 min. The PCR products were further evaluated using 2% agarose gel electrophoresis. Throughout the DNA extraction process, ultrapure water was used as the negative control to exclude the possibility of false-positive PCR results. The PCR products were purified using AMPure XT beads (Beckman Coulter Genomics, Danvers, MA, USA) and quantified using a Qubit (Invitrogen, USA). Amplicon pools were prepared for sequencing. The size and quantity of the amplicon library were assessed using an Agilent 2100 Bioanalyzer (Agilent, USA) and Library Quantification Kit for Illumina (Kapa Biosciences, Woburn, MA, USA), respectively. Libraries were sequenced on a NovaSeq PE250 platform.

### 2.5 Metagenomic sequencing

The experimental procedure used for metagenomic sequencing was based on previous studies (18, 19) with minor modifications. After sample clustering analysis, three samples with higher similarity were selected from each treatment. So, a total of 9 samples were used for the metagenomic sequencing. A DNA library was constructed using the TruSeq Nano DNA LT Library Preparation Kit (FC-121-4001). DNA was fragmented using dsDNA fragmentase (NEB, M0348S) by incubation at 37°C for 30 min. Library construction was initiated using the fragmented cDNA. Blunt-end DNA fragments were generated using a combination of fill-in reactions and exonuclease activity, and size selection was performed using sample purification beads. An A-base was then added to the blunt ends of each strand to prepare them for ligation with indexed adapters. Each adapter contained a T-base overhang to ligate the adapter to A-tailed fragmented DNA. These adapters contained the full complement of the sequencing primer hybridization sites for single-, paired-end, and indexed reads. The single- or dual-index adapters were ligated to the fragments, and the ligated products were amplified with PCR using the following conditions: initial denaturation at 95°C for 3 min, 8 cycles of denaturation at 98°C for 15 s, annealing at 60°C for 15 s, extension at 72°C for 30 s, and then final extension at 72°C for 5 min. Paired-end 2×150 bp sequencing was performed on an Illumina HiSeq 4000 platform (LC Sciences), based on the recommended protocol provided by the manufacturer.

### 2.6 Bioinformatic analysis

Raw sequencing reads were processed to obtain valid reads for further analyses. Sequencing adapters were removed from the sequencing reads using Cutadapt v1.9 (20). The low-quality reads were then trimmed using fqtrim v0.94 using a sliding-window algorithm, and the remaining reads were aligned to the host genome using bowtie2 (21) to remove host contamination. Once quality-filtered reads were acquired, they were *de novo* assembled to construct a metagenome for each sample, aided by IDBA-UD (22). All coding regions (CDS) of the metagenomic contigs were predicted using MetaGeneMark v3.26. The acquired CDS sequences of all samples were clustered using CD-HIT v4.6.1 (23) to obtain unigenes. The abundance of unigenes in each sample was estimated by TPM in accordance with the number of aligned reads using bowtie2 v2.2.0 (21). The lowest common ancestor taxonomy of the unigenes was obtained by alignment against the NCBI NR database using DIAMOND v 0.7.12 (24). Functional annotations of the acquired unigenes, including Gene Ontology (GO) and Kyoto Encyclopedia of Genes and Genomes (KEGG), were performed. Based on the taxonomic and functional annotations of the obtained unigenes, along with their abundance profiles, differential analysis was performed at each taxonomic, functional, or gene level using Fisher’s exact test (non-replicated groups) or the Kruskal–Wallis test (replicated groups).

## 3 Results

### 3.1 Data acquisition

**Figure 1:**
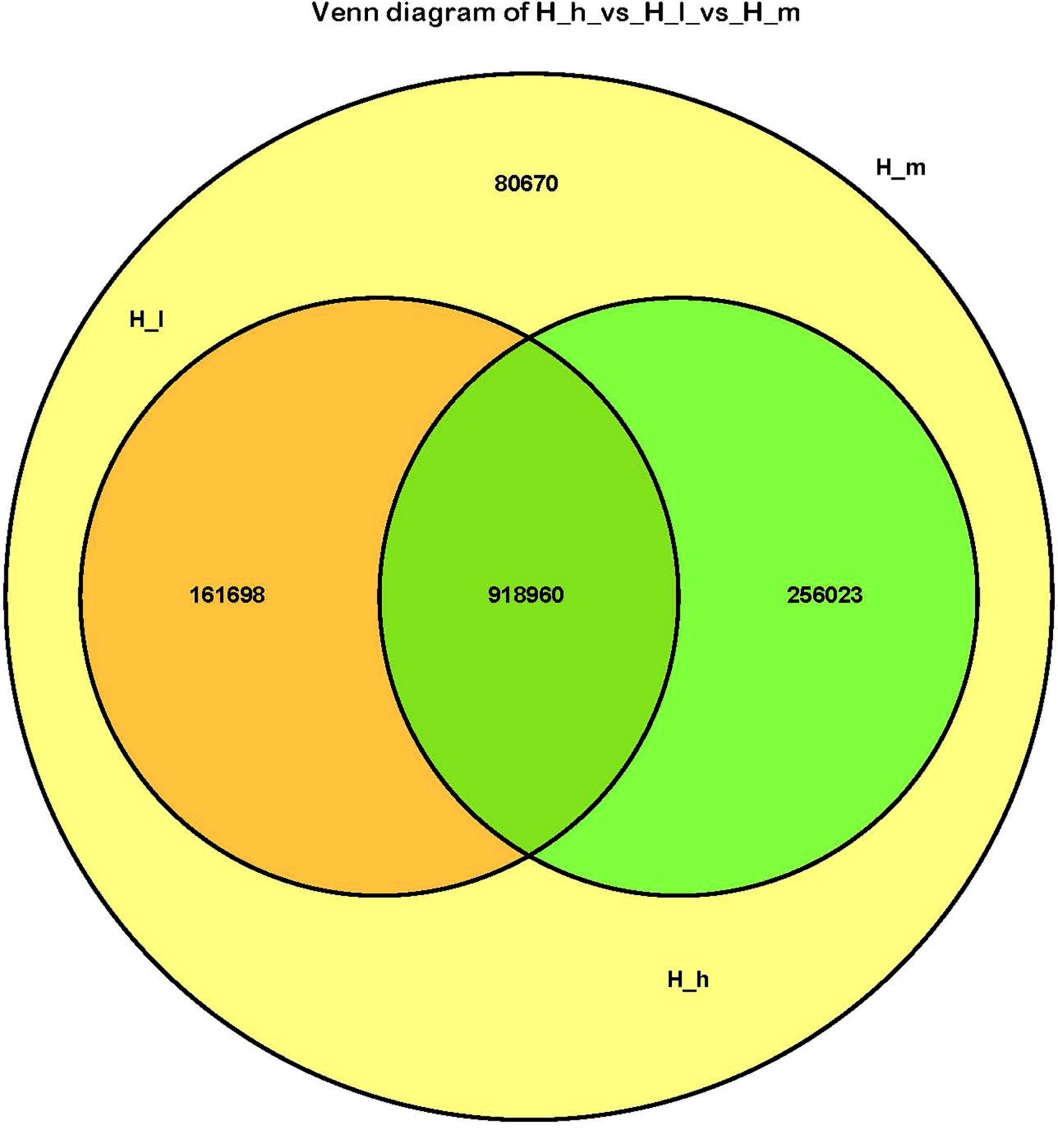
The Venn diagram of the H_I group *vs* the H_m group *vs* the H_h group. The orange circle represented the H_I group; the yellow circle represented the H_m group; the green circle represented the H_h group.

A total of 376,278,516 high-quality data points (clean data) were generated from the metagenomic sequencing of nine fecal samples. The acquisition rate of clean data was higher than 97%. Sequences longer than 500 nucleotides were used in the present study. The coding regions (CDS) of the assembled contigs were predicted using MetaGeneMark and contigs shorter than 100 nt were removed. CD-HIT was used to generate a nonredundant set and to output a cluster file (identity=95%, coverage=90%). The counts of each unigene were calculated using bowtie2 by mapping the reads of each sample onto the unigenes. The abundance of the obtained unigenes was estimated using TPM. In the present study, the total number of acquired unigenes was 1,417,351. Among these unigenes, the numbers of unigenes containing the start or stop codons were 359,225 (25.34%) and 263,828 (18.61%), respectively. The number of unigenes with the start and stop codons was 599,224 (42.28%). The remaining 195,074 (13.76%) unigenes did not have the start or stop codon. The total length of all acquired unigenes was 1009.34 Mbp, with an average length of 712 bp. A Venn diagram reflects the similarities among the three treatment groups (**Figure 1**). There were 918960 genes simultaneously present in both the H_h and H_I groups, and 256023 and 161698 genes were exclusively present in the H_h and H_I groups, respectively. In addition, all genes in the H_h group were present in the H_m group. 242368 genes were found to only be present in the H_m group. All genes in the H_I group were present in the H_m group. 336693 genes were exclusively found in the H_m group.

### 3.2 Annotation of bacterial species

**Figure 2:**
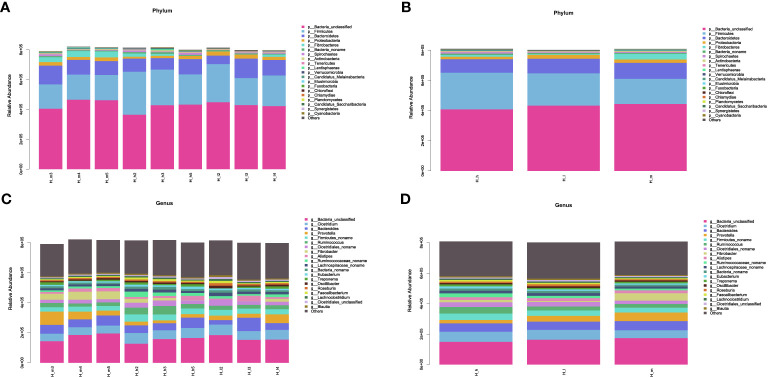
The stacked bar charts of bacterial abundance at the phylum **(A, B)** and genus **(C, D)** levels. The X-axis represented the nine samples used in this study **(A, C)** or the three treating groups **(B, D)**. The Y-axis represented bacterial abundance in each sample at the phylum **(A, B)** and genus **(C, D)** levels. The bacterial species were represented by different colors.

**Figure 3:**
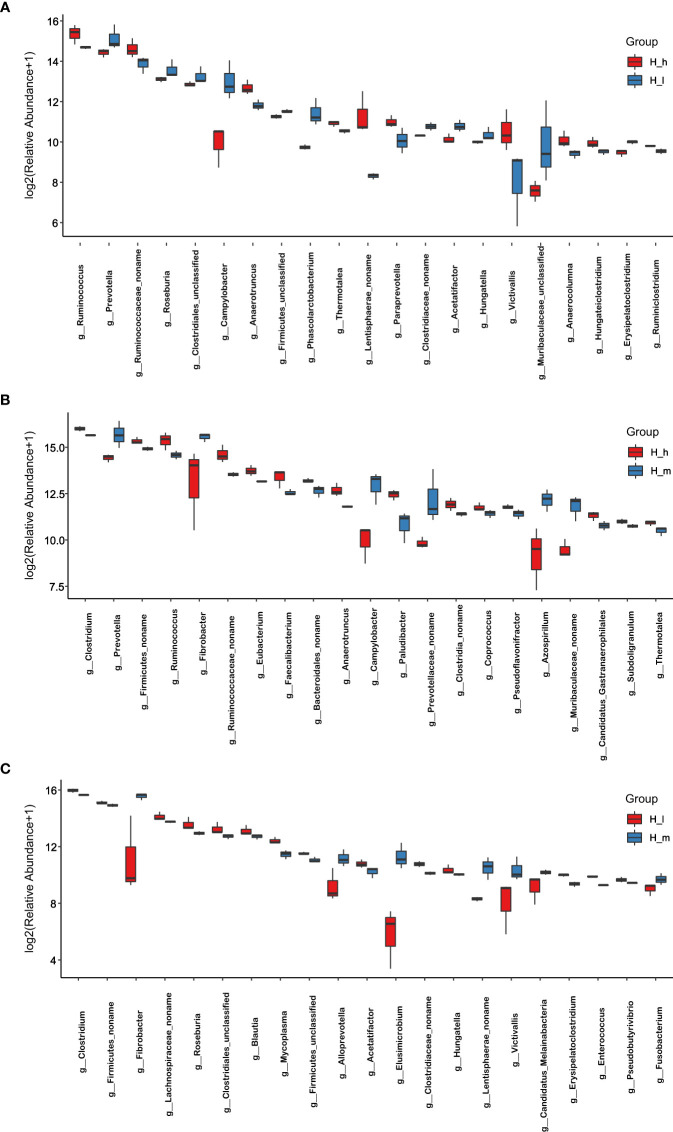
Comparison of bacterial abundance in the H_I, H_m, and H_h groups at genus level. The X-axil represented the bacterial genus. The Y-axil represented the abundance (log2 (Relative Abundance+1)). **(A–C)** represented the comparison between the H_h group and the H_I group, between the H_h group and the H_m group, and between the H_I group and the H_m group, respectively.

Based on the classification system of microbial species in the NCBI nr_meta database, the results of this study were revealed by Super Kingdom, Phylum, Class, Order, Family, Genus, and Species using the Lowest Common Ancestor algorithm. Detailed information associated with bacterial abundance at the phylum and genus levels is included in **Supplementary Tables S1–S4**. The most abundant 20 classifications were selected to produce stacked bar charts from Super Kingdom to Species in each sample. The results related to phylum and genus abundances are shown in **Figure 2**. **Figure 2A** shows the phylum abundance of each sample used, and **Figure 2B** shows the phylum comparison between the three treatments. Among the 146 identified phyla, the most abundant were *Firmicutes, Bacteroidetes, Proteobacteria*, and *Fibrobacteres*, as shown in **Figures 3A, B**. However, approximately 50% of the acquired sequences *(Bacteria_unclassified)* could not be classified into any known phyla. **Figure 2C** shows the genus abundance of each sample used, and **Figure 2D** shows the genus comparison among the three treatments in this study. After analysis, 2758 genera were identified. Among these genera, *Clostridium, Bacteroides, Prevotella, Firmicutes_noname, Ruminococcus, Clostridiales_noname*, and *Fibrobacter* were dominant (**Figures 2C, D**). However, approximately 20% of sequences (*Bacteria_unclassified)* could not be classified into any known genus.

### 3.3 Bacterial diversity analysis

**Figure 4:**
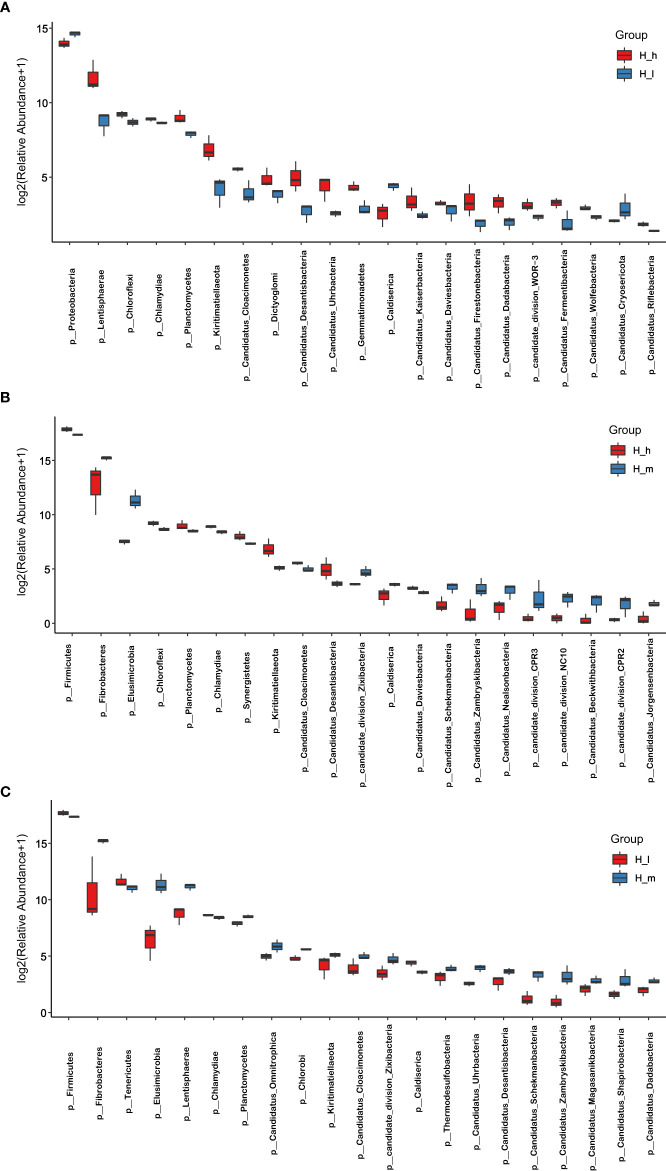
Comparison of bacterial abundance in the H_h, H_m, and H_I groups at phylum level. The X-axil represented the bacterial phyla. The Y-axil represented the abundance (log2 (Relative Abundance+1)). **(A–C)** represented the comparison between the H_h group and the H_I group, between the H_h group and the H_m group, and between the H_I group and the H_m group, respectively.

Indices, including Chao1, Observed species, and shannon, were used to analyze microbial species diversity in fecal samples collected from the three treatments. These data are presented in **Table 2**. The number of observed species in the H_m group was significantly higher than that in the H_I group (*P<0.01*). However, no dissimilarity was found between the H_h and the H_m or H_I groups (*P>0.05*). Similarly, the chao1 value in the H_m group was significantly higher than that in the H_I group (*P<0.01*). However, no difference was observed between the H_h and the H_m or H_I groups (*P>0.05*). In terms of the Shannon value, as shown in **Table 2**, the Shannon value in H_m group was significantly less than that in H_h group (*P<0.05*). However, the Shannon index value in the H_I group did not differ from those in the other two groups (*P>0.05*). Based on the bacterial abundance presented in each sample, the Mann–Whitney U test was used to determine the differences in abundance at the phylum and genus levels. The results related to the different bacteria at the phylum level are shown in **Figure 4**. The abundance of *Proteobacteria, Caldiserica*, and *Candidatus_Cryosericota* in the H_h group was lower than that in the H_I group (**Figure 4A**). In contrast, phyla, such as *Lentisphaerae, Chloroflexi, Chlamydiae, Planctomycetes*, and *Kiritimatiellaeota*, dominated in the H_h group. The results of comparison between the H_m and H_h groups are shown in **Figure 4B**. Compared to the H_m group, these phyla, including *Firmicutes*, *Chloroflexi, Planctomycetes, Chlamydiae*, and *Synergistetes*, were significantly enriched in the H_h group. However, other phyla, such as *Fibrobacteres*, *Elusimicrobia, Caldiserica, Candidate_division_Zixibacteria*, *Candidatus_Daviesbacteria, Candidatus_Schekmanbacteria*, and *Candidatus_Zambryskibacteria*, were more abundant in the H_m group. As shown in **Figure 4C**, *Fibrobacteres*, *Elusimicrobia*, *Lentisphaerae, Planctomycetes, Candidatus_Omnitrophica, Chlorobi*, and *Kiritimatiellaeota* were dominant in the H_m group compared to those in the H_I group. *Firmicutes*, *Tenericutes, Chlamydiae*, and *Caldiserica* were enriched in the H_I group. At the genus level, the results are shown in **Figure 3A**; the abundance levels of these genera in the H_h group, such as *Prevotella, Roseburia, Clostridiales_unclassified, Campylobacter, Firmicutes_unclassified*, and *Phascolarctobacterium*, were lower than those in the H_I group. However, *Ruminococcus, Ruminococcaceae_noname, Anaerotruncus, Thermotalae, Lentisphaerae_noname* and *Paraprevotella* were significantly more abundant in the H_h group. A comparison of the H_h and H_m groups is presented in **Figure 3B**. *Clostridium, Firmicutes_noname, Ruminococcus, Eubacterium, Faecalibacterium, Bacteroidales_noname*, and *Anaerotruncus* were more abundant in the H_h group than in the H_m group. However, *Prevotella, Fibrobacter, Campylobacter*, *Prevotellaceae_nonname*, and *Azospirillum* were dominant in the H_m group. In addition, as shown in **Figure 3C**. *Fibrobacter, Alloprevotella, Elusimicrobium, Victivallis, Lentisphaerae*, and *Fusobacterium* were less abundant in H_I than in H_m. *Clostridium, Firmicutes, Lachnospiraceae_noname, Roseburia, Clostridiales_unclassified, Blautia, Mycoplasma*, and *Hungatella* were enriched in the H_I group.

**Table 2:** The values related to the observed_species, Shannon, and chao 1 index.

Treatment groups	observed_species	Shannon	chao1
H_I	10338 ± 181^b^	8.19 ± 0.16	10856.38 ± 109.28^b^
H_m	13275 ± 1529^a^	8.09 ± 1.46^a^	14008.64 ± 1723.11^a^
H_h	12120 ± 555	8.41 ± 0.44^b^	12545.57 ± 432.40

Values in the same row with different superscripts (a and b) indicate significant differences among the groups (P <0 .05).

### 3.4 Functional annotation

**Figure 5:**
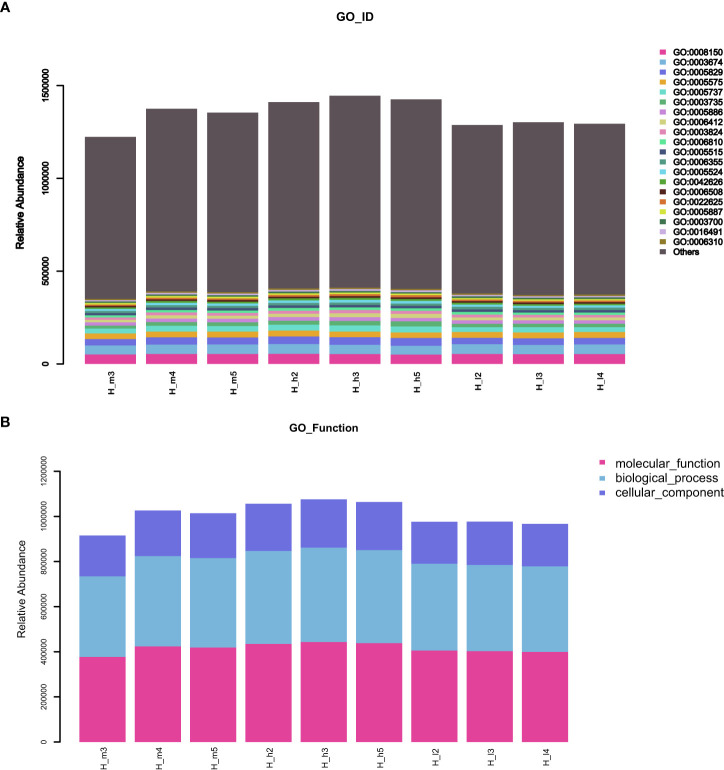
The stacked bar charts of GO functional annotation of the obtained unigenes. **(A)** represented the GO_ID functional enrichment of the acquired unigenes. **(B)** represented the GO functional annotation of the acquired unigenes in the samples. The X-axis represented the individual sample. The Y-axis represented the relative abundance.

**Figure 6:**
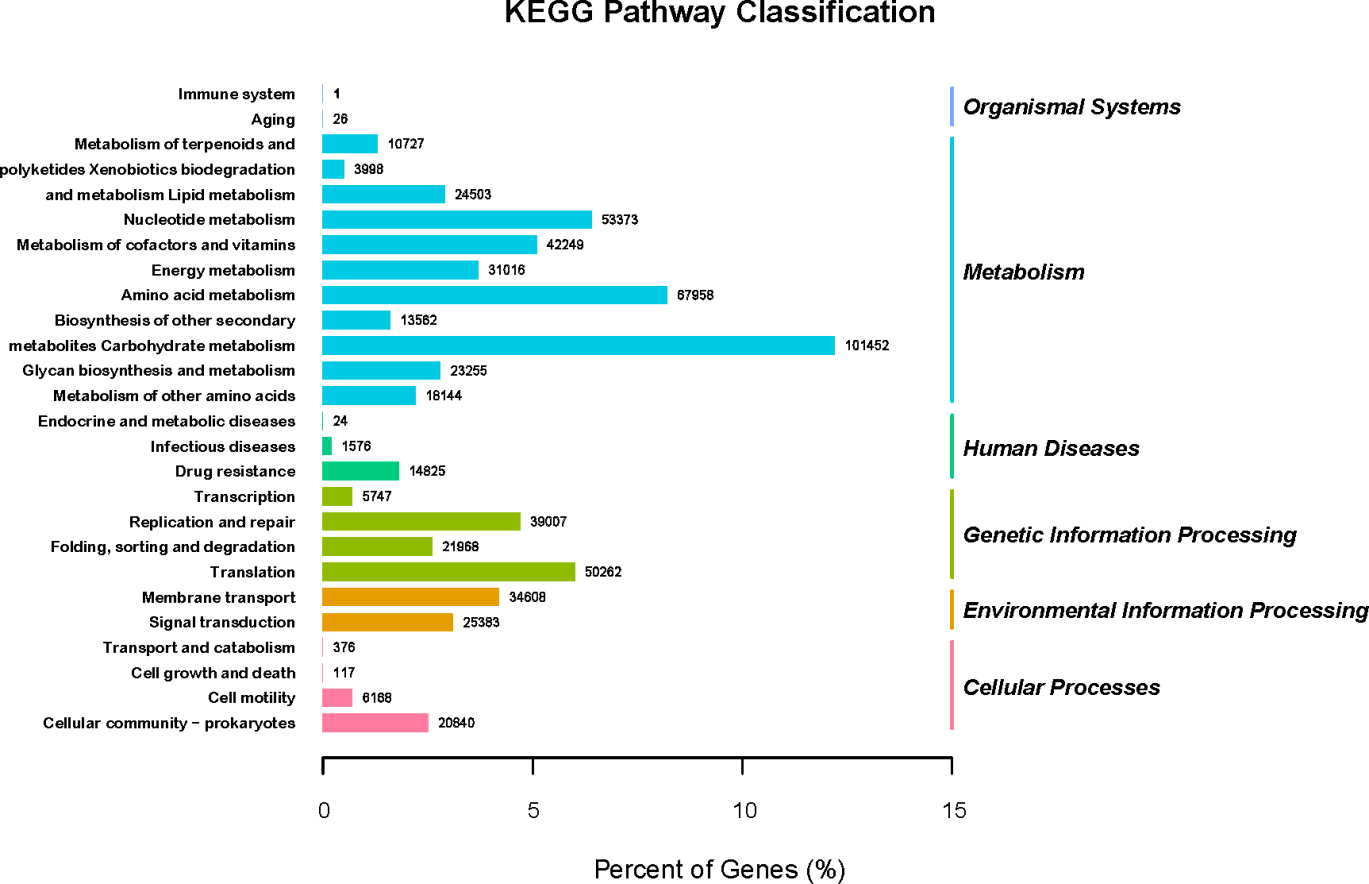
The KEGG pathway classification of the acquired genes. The X-axel represented the percentage of genes. The left Y-axis represented the involved KEGG pathways (KEGGLevel2). The right Y-axis represented the KEGGLevel1 functional classification. The digit on each column was the number of involved genes.

**Figure 7:**
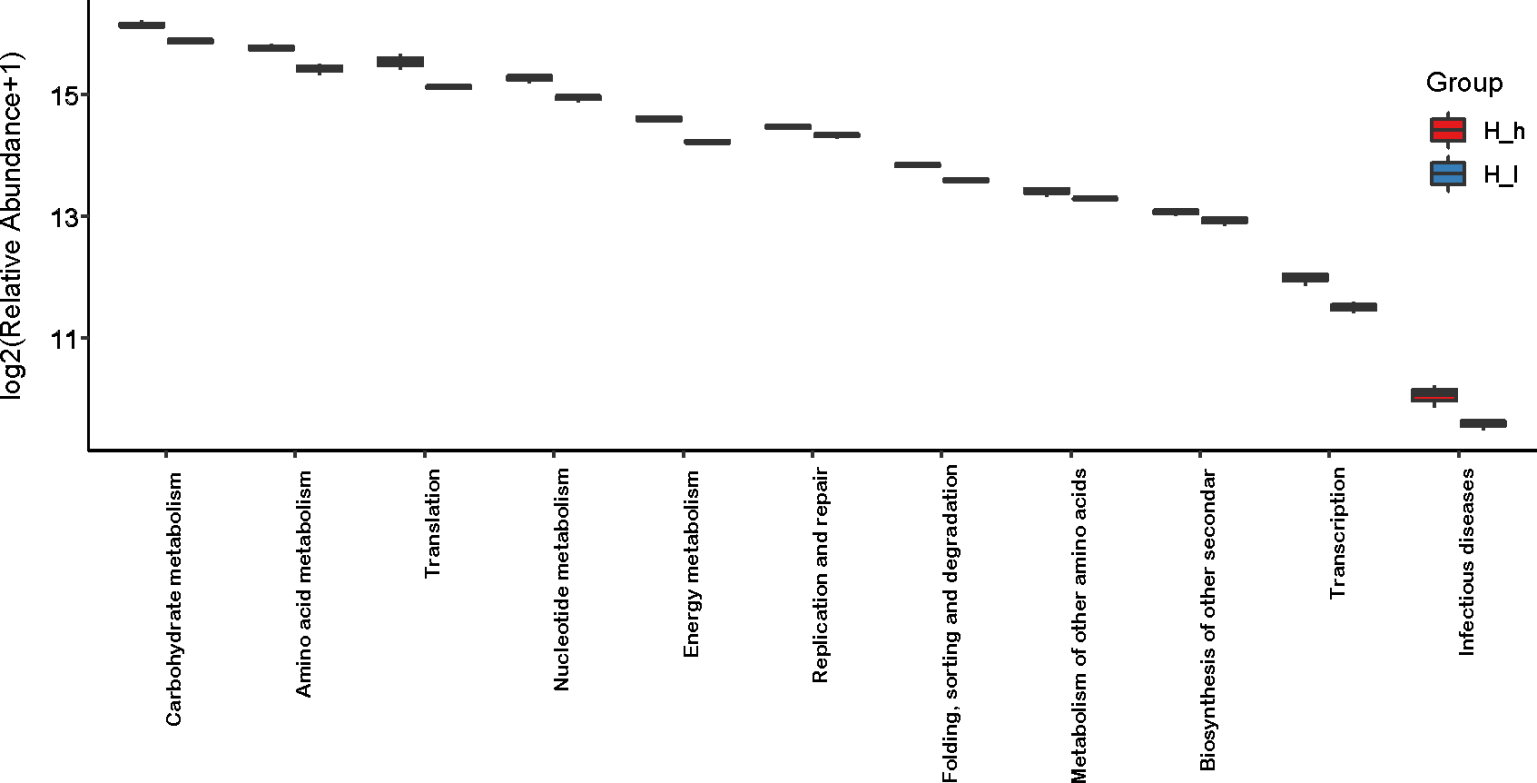
Comparison of abundance of genes involved in KEGG pathways in the H_h group and the H_I group. The X-axis represented the involved pathways. The Y-axis represented the abundance (log2 (Relative Abundance+1)). The red box represented the H_h group. The blue box represented the H_I group.

GO (http://www.geneontology.org) is a major bioinformatics initiative used to unify the representation of genes and gene product attributes across all species. As shown in **Figure 5A**, most of the identified unigenes were clustered in biological process (GO:0008150), molecular function (GO:0003674), cytosol (GO:0005829), cellular component (GO:0005575), cytoplasm (GO:0005737), structural constituent of ribosome (GO:0003735), plasma membrane (GO:0005886), translation (GO:0006412), and catalytic activity (GO:0003824). Most of the unigenes were enriched in molecular functions and biological processes, as shown in **Figure 5B**. In addition, as shown in **Supplementary Figure S1**, the abundance of genes involved in molecular functions, biological processes, and cellular components was significantly higher in the H_h group than in the H_I group. In addition, as shown in **Supplementary Figure S2**, a similar pattern of change was observed between the H_h and H_m groups. However, no significant differences were observed between the H_m and H_I groups. The KEGG annotation of the unigenes was performed. As shown in **Figure 6**, unigenes were found to be primarily involved in organism systems, metabolism, human diseases, genetic information processing, environmental information processing, and cellular processes. Among the unigenes, 101452, 67958, 53373, 42249, and 31016 may play roles in carbohydrate, amino acid, nucleotide, cofactor, vitamin, and energy metabolism. Since this study showed that high-protein diets benefited the growth of lactating ewes compared to low-protein diets, the KEGG functional differences between the H_h and H_I groups were analyzed. In the H_h group, the unigenes involved in carbohydrate metabolism, amino acid metabolism, nucleotide metabolism, energy metabolism, metabolism of other amino acids, and biosynthesis of other secondary metabolites were more abundant than those in the H_I group (**Figure 7**). Furthermore, an interesting phenomenon was that in comparison with the H_I group, more genes in the H_h group were found to be potentially associated with infectious disease.

### 3.5 Gene expression differences and enrichment analysis

**Figure 8:**
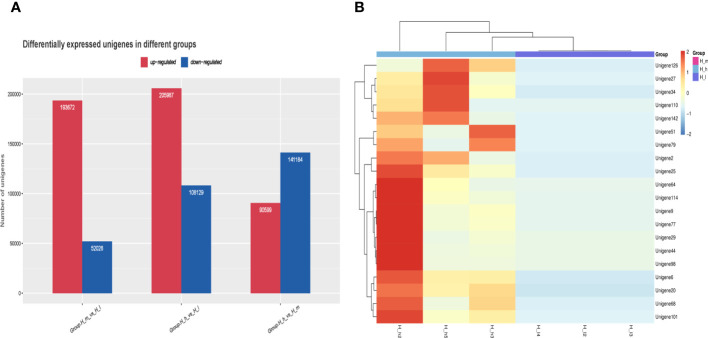
Differentially expressed unigenes between the treatments fed with different levels of proteins **(A)** and the cluster analysis of differential gene expression levels **(B)**. Red color represented high enrichment. Blue color represented low enrichment.

**Figure 9:**
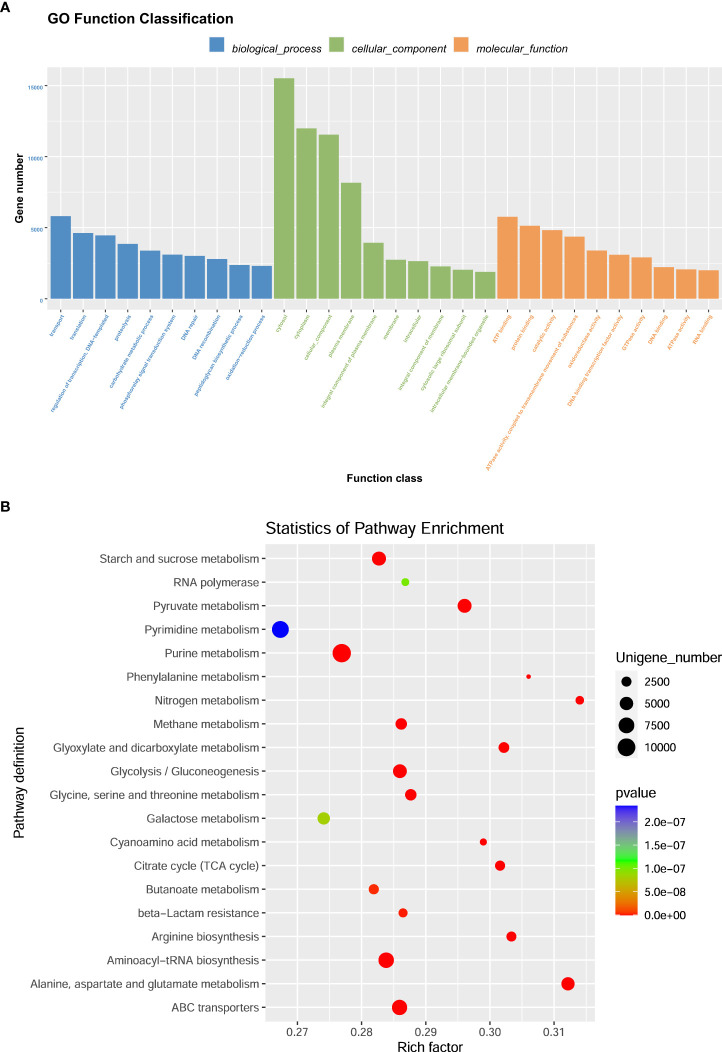
The enrichment analysis of GO function of differentially expressed genes. **(A)** represented that the genes were classified into biological processes, cellular components and molecular function. The bar represents the number of differentially expressed genes. **(B)** represented that the scatter plot of differentially expressed gene GO enrichment. The *P* value was calculated using the Fisher’s exact test. The X-axis represents the rich factor, and the Y-axis means the KEGG pathway. Each bubble refers to the number of unigenes. Rich factor means the number of differentially expressed genes located in the KEGG/the total number of genes located in the KEGG.

DEGs were the most valuable results of metagenomic sequencing. These results represent the differential expression of genes among different treatments or samples. In general, the default threshold of genes with a significant difference is |log2 (fold)_ change)|≥1 and *P* value less than 0.05. Compared to those in the H_I group, 193672 and 205987 unigenes were upregulated in the H_m and H_h groups, respectively (**Figure 8A**). 52026 and 108129 genes were separately enriched in the H_I group. 90599 genes were more abundant in the H_h group than in the H_m group, and 141184 genes were enriched in the H_m group. Functional enrichment analysis of DEGs was performed. The most abundant 20 genes (**Figure 8B**) were selected to produce a heatmap illustrating the clustering pattern of DEGs between the H_h and H_I groups. We observed that the degree of enrichment of these genes was greater in the H_h group than in the H_I group. The GO functional classification of DEGs between the H_h and H_I groups is shown in **Figure 9A**. In terms of biological processes, the DEGs were primarily involved in transport, translation, and carbohydrate metabolic processes. The identified DEGs were mainly present in the cytosol and cytoplasm. Regarding molecular functions, most DEGs were involved in ATP binding, protein binding, and catalytic activity. The scatter plot of KEGG enrichment (**Figure 9B**) demonstrated that the DEGs were mainly responsible for metabolic activities, such as alanine, aspartate, and glutamate metabolism; arginine biosynthesis; citrate cycle; nitrogen metabolism; pyruvate metabolism; starch and sucrose metabolism; glycolysis; and methane metabolism.

## 4 Discussion

The lactating stage is critical for ewes and their lambs, as ewes require essential nutrition to support themselves and their lamb’s survival and health at this stage. The addition of proteins to daily diets improves the metabolic activity of ruminants (2, 3, 5) In the present study, we analyzed the effects of dietary proteins on the bacterial characteristics in fecal samples using 16S rDNA sequencing and metagenomic approaches. Our findings demonstrate that dynamic changes in fecal bacteria and their genes are strongly influenced by the levels of dietary proteins. The utilization of dietary proteins by ruminants is closely associated with the involvement of gastrointestinal bacteria. Previous studies have primarily focused on rumen-derived bacteria (4–8, 10, 11). However, some researchers have switched their attention to fecal bacteria because of the potential relationship between fecal and rumen bacteria (13–15, 17).

In the present study, the richness of genes in the H_m group was the highest. All genes present in the H_h and H_I groups were included in the H_m group. Notably, the level of dietary proteins in the H_m group represented a regular protein level in the ewes’ daily diet during the lactation stage. However, it may lead to a reduction in the types of acquired genes in the feces. Therefore, the maintenance of normal dietary protein levels benefits the richness of fecal bacterial communities. In addition, there are 918960 genes were simultaneously present in all three treatments, demonstrating that these genes are highly conserved and essential for the metabolic activities of ewes. They may also play important roles in ruminant gut microbial ecology. A total of 146 phyla were identified in this study. *Firmicutes* and *Bacteroidetes* were the dominant phyla in all fecal samples, regardless of the dietary protein levels.

Our findings are consistent with other reports on sheep (10, 17), cattle (7, 14, 15, 25), pigs (26–29), goats (30), and humans (31, 32). *Firmicutes* and *Bacteroidetes* have been found to constitute the majority of the gut-derived phylotypes in mammalian species (33–36), suggesting that *Firmicutes and Bacteroidetes* play critical roles in the microbial ecology of ruminant guts. Our study further demonstrated that *Firmicutes* was the most abundant phylum, with an abundance of 20%-30% out of the total bacteria obtained. The dominance of *Firmicutes* further supports the conclusions of previous studies on sheep and cattle (14, 17, 35, 37). However, according to the report of Tanca et al., *Firmicutes* and *Bacteroidetes* account for 80% of total bacteria obtained in sheep feces (17). In this study, the ratio of *Firmicutes* to *Bacteroidetes* was approximately 40%. In addition, according to a report by Kim et al., the ratio of *Firmicutes* was over 50% in bovine fecal samples (15), and greatly higher than the ratio of *Firmicutes* in ewes’ feces in this study. In contrast, the most abundant phylum detected in goat rumen was *Bacteroides*, not *Firmicutes*, which accounted for an average 42.11% of the total bacterial community (30). Furthermore, the bacterial characteristics of sheep feces differ from those of pig and chicken feces. In a previous study, *Bacteroidetes* was predominant in pig fecal samples at their different growth stages, with a mean relative abundance ranging from 42.0%–51.9%, followed by *Firmicutes* (29). In chickens, *Proteobacteria* (38.9%) were dominant, followed by *Firmicutes* (36.4%), *Bacteroidetes* (15.8%), and *Tenericutes* (8.9%) (38).

The differences between the abovementioned studies may be due to specific factors, such as animal species, growth stage, sampling position, and dietary components used. Based on this study, as the third most abundant phylum, the richness of *Proteobacteria* in the H_h group (2.03%) was slightly lower than those in the H_m group (2.77%) or the H_I group (3.15%). *Proteobacteria* includes a wide variety of pathogenic bacteria (30). Therefore, increasing the levels of dietary proteins may improve the capability of disease resistance in lactating sheep. In pigs, Zhao et al. found that *Proteobacteria* showed a significant decline along with age (39). As neonatal pigs are more susceptible to diseases, there may be an inverse relationship between the abundance of *Proteobacteria* and disease resistance. Another piece of evidence is the use of antimicrobial peptides in goat daily diets (30). Stackebrandt et al. found that *Proteobacteria* were less abundant in groups supplemented with AMPs (antimicrobial peptides) (30). In chickens infected with multidrug-resistant (MDR) *Escherichia coli*, the most abundant phylum in the feces was *Proteobacteria (* 38). These studies further demonstrate that the abundance of *Proteobacteria* may be negatively associated with disease sensitivity. In this study, the abundance of *Bacteroidetes* in the H_h group was slightly lower than that in the H_m and H_I groups, which agrees with a previous report showing that high-producing cows have a lower abundance of *Bacteroidetes (* 40). However, in contrast to a previous study on cows, *Planctomycetes, Synergistetes, and Chloroflexi* were significantly enriched in the H_h group in this study. However, the roles of these phyla require further investigations. At the genus level, 2758 genera were identified. Among these genera, *Clostridium, Bacteroides, Prevotella, Firmicutes_noname, Ruminococcus, Clostridiales_noname*, and *Fibrobacter* were the most dominant. Approximately 20% sequences (*Bacteria_unclassified)* could not be classified into any known genus.

Here, we found that *Prevotella* was not the most dominant genus in sheep fecal samples, which is different from the genus-level characteristics in goat rumen (41). As a permanent resident of the bacterial community in the mature rumen, *Prevotella* is assumed to comprise a large part of the rumen microbial genetic and metabolic diversity. According to the report of Ren et al., at the genus level, *Prevotella* dominated the assignable sequences, on average accounting for 29.21% of the total bacteria (30), which may correlate with the fact that most of digestive activities of fibers are conducted in rumen. However, in the present study, a lower abundance of *Prevotella* was observed in the feces of ewes fed a high-protein diet than in the H_I and H_m groups, demonstrating the negative effects of a high-protein diet on the abundance of *Prevotella*. In another study, the growth of calves, the abundance of *Prevotella* gradually increased with calf growth. Compared to that in newborn calves, *Prevotella* showed an almost 500-fold increase in the adult rumen (42, 43). Also, *Prevotella* is widely associated with plant polysaccharide digestion and are enriched in plant polysaccharides (44). According to a report by Jami and Mizrahi, three ruminal species of the pivotal *Prevotella* genus account for up to 70% of the rumen bacterial population (43). These species can utilize starches, non-cellulosic polysaccharides, and simple sugars as energy sources, and succinate is the major fermentation end-product (41). In addition, in newborn calves (1- and 3-day old), the main genus in their rumen was *Bacteroides*, whereas in the older age groups, the main ruminal genus was almost exclusively *Prevotella (* 25). Newborn calves are primarily dependent on milk, which is rich in proteins (42, 43). However, along with their growth, the ratio of plant-derived components in their daily diets gradually increased. *Prevotella* seems to be positively associated with plant-derived diets. In addition, a similar compositional alteration in the *Prevotella* genus was reported in a previous study that compared the gut microbiota of children from Europe and rural Africa (45). In that study, the genus *Prevotella* dominated the *Bacteroidetes* phylum, accounting for 53% of the total gut bacteria in African children. In contrast, *Bacteroides* was the primary genus found in European children. Moreover, a much higher ratio of the *Bacteroidetes* phylum was observed in African children, along with a lower abundance of *Firmicutes (* 45). These differences may have been caused by the dietary composition ingested by these children. The African diet is mainly composed of plant fiber, whereas the European diet is rich in animal protein, sugar, starch, and fat (but low in plant fiber) (45).

Similarly, *Prevotella* was not the main genus in fecal samples from neonatal pigs from days 3 to 7. However, *Prevotella* became dominant between days 14 and 35 (29). All these studies demonstrate that *Prevotella* plays an essential role in the mammals’ growth and metabolic activities. *Prevotella* produces acetate, which can be further transformed into butyrate with the aid of some butyrate-producing bacteria (44, 46). Approximately 50% of butyrate-producing bacteria can produce butyrate from acetate (47). These metabolic and biosynthetic activities may further improve intestinal barrier function and reduce inflammation in the mammalian gut (48, 49).

When the sheep were fed a high-protein diet, another genus that showed an evident increase in fecal samples was *Ruminococcus*. *Ruminococcus* generally comprises cellulolytic bacteria that are commonly present in the adult rumen (50). However, at the genus level, the rumen fluid of high-production cows was significantly depleted of *Ruminococcus (* 39). In general, the low-production cows were fatter than the high-production cows. Thus, *Ruminococcus* has been suggested to be negatively associated with milk production (51), implying a positive relationship between *Ruminococcus* and cow meat production. Another study assessed the effects of probiotics on gut microbiota and their association with childhood obesity (52). The results revealed an abundant increase in the genus *Ruminococcus* in overweight participants after intervention with probiotics compared to normal-weight participants (52).

In the present study, *Fibrobacter* was more abundant in the H_h and H_m groups than in the H_I group, implying a relationship between *Fibrobacter* and the growth of ewes. *Fibrobacter* is a cellulose-degrading microbes (53, 54). The fermentation products of *Fibrobacter* are acetate, propionate, and succinate (30). Acetate, propionate, and butyrate were the major volatile fatty acids, accounting for 95% of the total volatile matter content in rumen (55). Volatile fatty acids, as the end-products of fermentation by rumen microbiome, provide 70%–80% of the calorific requirements for ruminants (56). Therefore, the improved growth performance of ewes fed high-protein diets may be due to an increase in volatile fatty acids (30). Wang et al. reported that ruminal infusion of soybean peptide increased the concentrations of ammonia, propionate, and volatile fatty acids, and improved nutrient digestion and ruminal fermentation in Luxi Yellow cattle (57). Another study indicated that feeding a higher-fiber diet increases the abundance of *Fibrobacter (* 43). In combination with the results of the present study, we further confirmed that increasing dietary protein levels benefits the digestion and usage of plant-derived fibers. *Fibrobacter* may play an important role in this process.

Currently, metagenomic technology is used to observe the relationship between fecal bacterial composition and clinical diseases in humans (58). Studies on other mammals have mainly focused on pig (59, 60) and cattle (61). However, studies on sheep are limited. In a previous study, 2097 gene families were identified in sheep feces, and the sheep fecal microbiota was found to be primarily involved in catabolism (17). The fecal microbiota metagenome was rich in genes associated with the membrane transport of molecules (ABC transporter, ATPase, permease, and SecA), DNA replication and repair (helicase, DNA polymerase, topoisomerase, and MutS), transcription (RNA polymerase), translation (tRNA synthetases and translation factors), and protein folding (chaperones), along with a few genes encoding metabolic enzymes (17). In goats, metagenomic technology has been used to investigate carbohydrate-active enzymes in the intestinal tract, revealing a low abundance of enzymes that target xylan and cellulose. Therefore, it may be concluded that plant cell-wall digestion does not occur in the intestinal tract and may mainly occur in the rumen (18).

Most of the bacterial genes obtained in the present study were clustered in biological processes, molecular functions, cytosol, cellular components, cytoplasm, structural constituents of ribosomes, plasma membranes, translation, and catalytic activity. In the H_h group, genes involved in molecular function, biological processes, and cellular components were more abundant than those in the H_I group, implying that increasing dietary protein levels maintained the physiological functions of gut bacteria better. Subsequently, in accordance with the KEGG results, the acquired genes were found to be primarily involved in organism systems, metabolism, human diseases, genetic information processing, environmental information processing, and cellular processes. Furthermore, several genes may influence the metabolic activities of carbohydrates, amino acids, nucleotides, and vitamins. The bacteria present in sheep feces are widely involved in the digestion and use of nutritional components, which is in agreement with a report by Tanca et al. In their study, several genes in sheep feces encoded metabolic enzymes (17). In this study, the abundance of genes involved in carbohydrate, amino acid, nucleotide, energy, and other amino acid metabolism was significantly higher in the H_h group than in the H_I group.

One of the main objectives of this study was to observe the differences in bacterial gene expression between these treatments. DEGs can also act as potential markers to evaluate productive traits in ruminants. As shown in **Figure 8**, when fed a high-protein diet, 205987 microbial genes were upregulated in sheep feces, and 108129 genes were enriched in ewes fed a low-protein diet. The heatmap results further demonstrated a completely different clustering pattern of DEGs between the H_h and H_I groups, confirming that the levels of dietary proteins greatly influenced bacterial gene characteristics in sheep feces. GO annotation indicated the roles of these DEGs in transport, translation, carbohydrate metabolic processes, ATP binding, protein binding, and catalytic activity. Additionally, the KEGG results indicated that these DEGs were mainly involved in metabolic activities such as amino acid metabolism, arginine biosynthesis, citrate cycle, nitrogen metabolism, pyruvate metabolism, starch and sucrose metabolism, glycolysis, and methane metabolism. Differing from the results of a study on pig (60), in this study, the ratio of the DEGs functioning in carbohydrate metabolic activity in sheep fecal metagenome was less than 2%. On the contrary, carbohydrate metabolism is the most abundant subsystem, representing 13% of pig fecal metagenome (60).

In conclusion, as an important nutritional resource, dietary proteins and their metabolism are closely associated with gut bacteria. However, further studies on the characteristics of the gut bacterial communities at different growth stages are required. To the best of our knowledge, the present study is the first attempt to assess the fecal bacterial characteristics of sheep during early lactation. Based on this study, we conclude that increasing dietary protein levels can enhance the weight gain of lactating ewes by modulating fecal bacterial composition. *Firmicutes* and *Bacteroidetes* were identified as the dominant phyla in the fecal samples, regardless of the protein levels used. 314116 DEGs were identified between the H_h and H_I group using metagenomic sequencing. These genes are primarily involved in metabolic activities. Notably, this study is preliminary and requires further in-depth analysis of the obtained data. Nevertheless, this study provides an opportunity to understand the fecal bacterial characteristics of lactating ewes, which may be helpful in developing a suitable dietary formula for optimizing the health of ewes and their lambs.

## Data availability statement

The datasets generated and/or analyzed during the current study are available in the NCBI repository. The accessions for 16S rDNA included SAMN25981821, SAMN25981822, SAMN25981823, SAMN25981824, SAMN25981825, SAMN25981826, SAMN25981827, SAMN25981828, SAMN25981829, SAMN25981830, SAMN25981831, SAMN25981832, SAMN25981833, SAMN25981834, SAMN25981835, SAMN25981836, SAMN25981837, SAMN25981838. The accessions for the metagenomic analysis included SAMN25981067, SAMN25981068, SAMN25981069, SAMN25981070, SAMN25981071, SAMN25981072, SAMN25981073, SAMN25981074, SAMN25981075.

## Ethics statement

In the present study, all experiments trials were approved by the ethical committee of the Yunnan Animal Science and Veterinary Institute (201911004). Moreover, all authors strictly followed the approved protocols and guidelines by the State Science and Technology Commission of the People’s Republic of China, 1988 and the Standing Committee of Yunnan Provincial People’s Congress 2007.10). Written informed consent was obtained from the owners for the participation of their animals in this study.

## Author contributions

JL and CLv: Sampling, Investigation, Methodology, Software, Validation, Data curation, Formal analysis, Writing - original draft. HY and BD: Data curation, Formal analysis, Investigation. XZ and XN: Animal management. SA, CLi, and YW: Revision, Writing - review and editing GQ: Conceptualization, Funding acquisition, Project administration, Resources, Investigation, Supervision, Visualization, Writing - review and editing. All authors contributed to the article and approved the submitted version.
