# Supplementary material for: Dietary protein levels modulate the gut microbiome composition through fecal samples derived from lactating ewes
Source: Front Endocrinol (Lausanne). 2023 Aug 9;14:1194425. doi: 10.3389/fendo.2023.1194425 (PMC10446493; doi:10.3389/fendo.2023.1194425)
Supplement: Supplementary file 1 [file DataSheet_1.docx]

Supplementary Material

**Dietary protein levels modulate the gut microbiome composition through fecal samples derived from lactating ewes**

First Author*, Co-Author, Co-Author

**Jiachong Liang ^1^*, Chunrong Lv ^1^*, Sikandar Ali ^2^, Hongyuan Yang ^1^, Xiaoqi Zhao ^1^, Xiaojun Ni ^1^, Chunyan Li ^1^, Baiji Danzeng^1^, Yajing Wang ^3^.**

*** Correspondence:** Guobo Quan: email: waltq20020109@163.com.

# Supplementary Figures and Tables

## Supplementary Figures

**Supplementary figure 1**: the abundance of differential expressed genes between the H_h group and the H_l group engaged on molecular function, biological process, and cellular component.
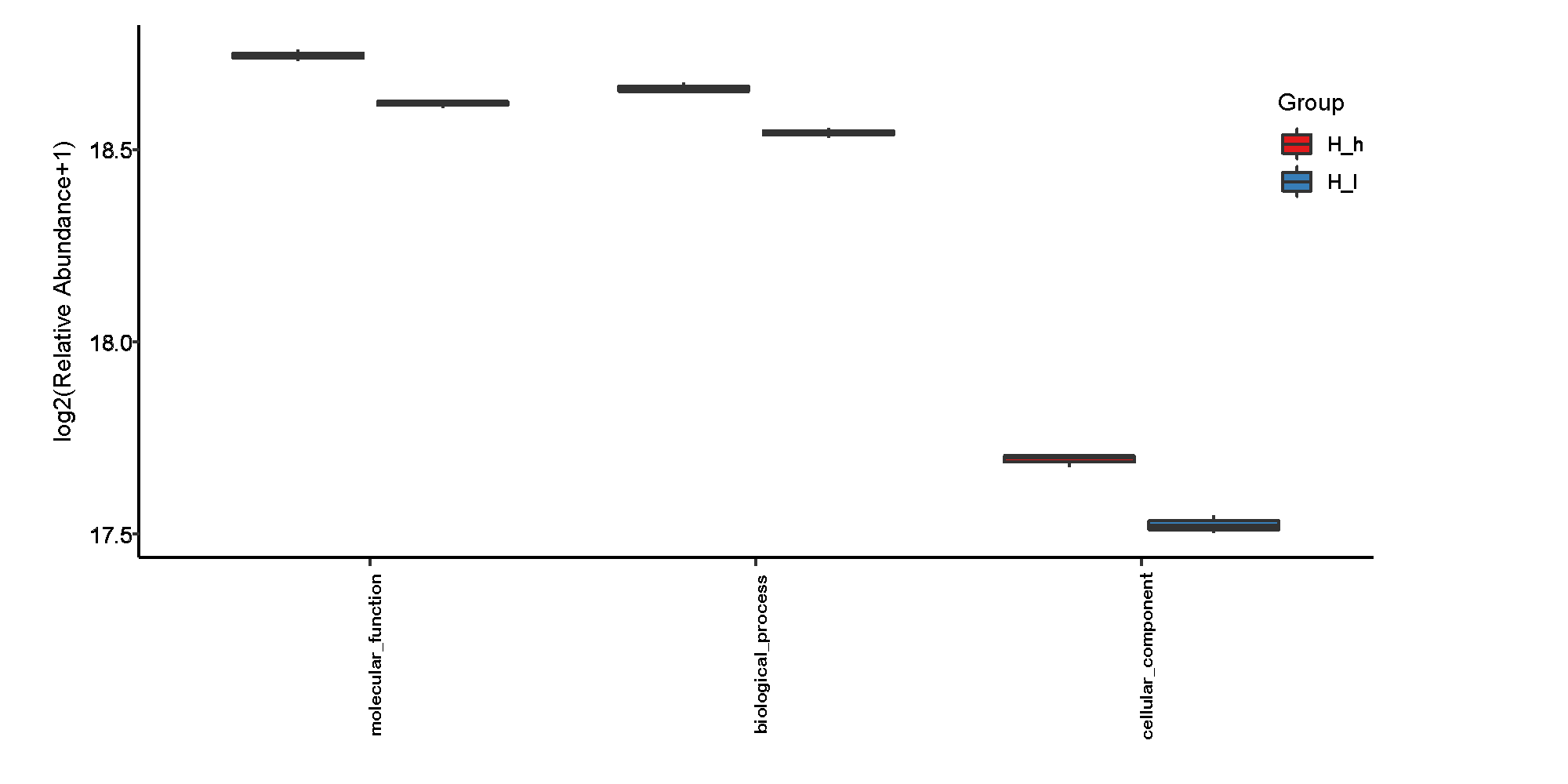


**Supplementary figure 2**: the abundance of differential expressed genes between the H_h group and the H_m group engaged on molecular function, biological process, and cellular component.


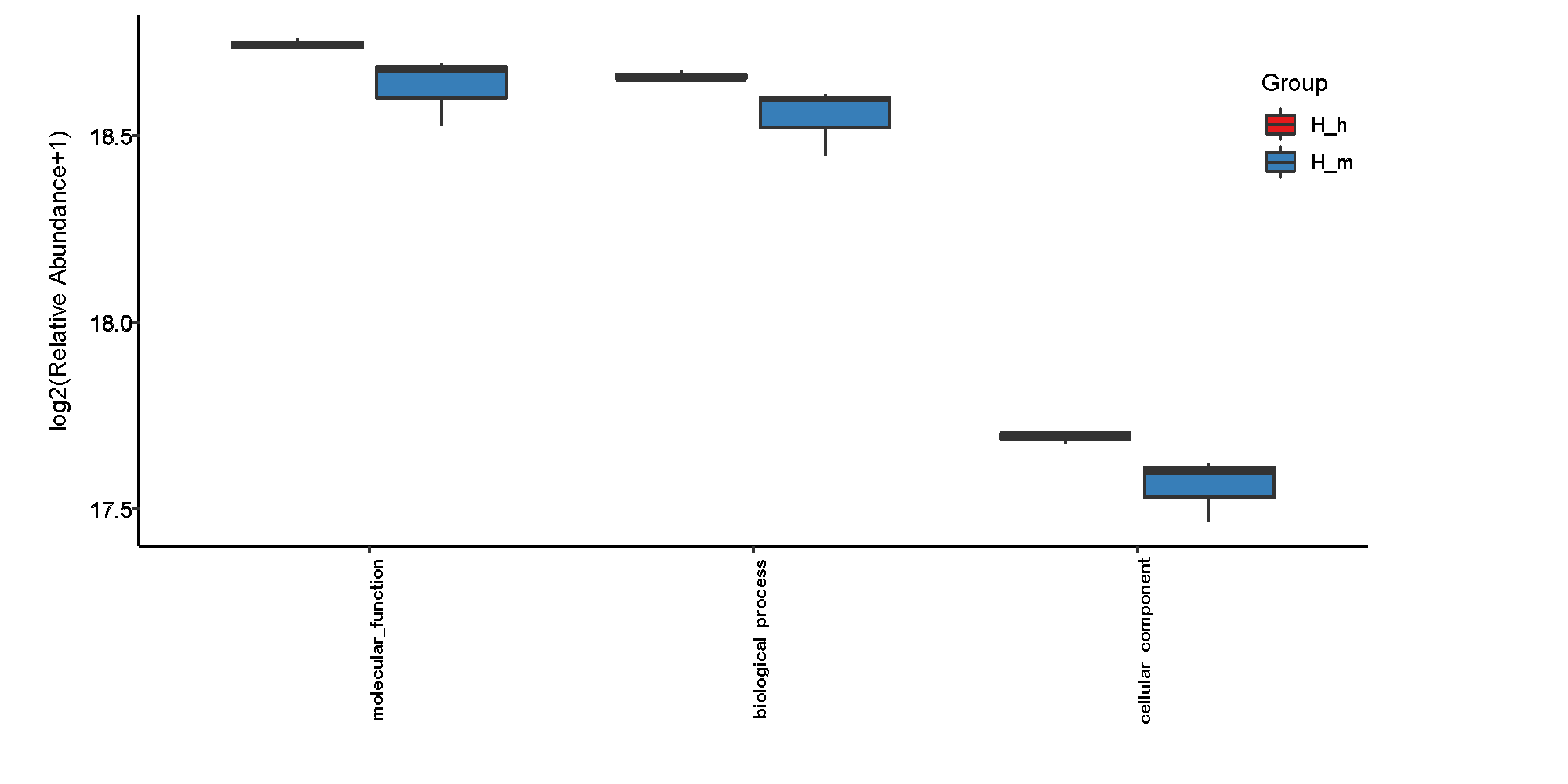


**Supplementary table 1:** the bacterial abundance at the phylum level in each sample.

| Phylum | H_m3 | H_m4 | H_m5 | H_h2 | H_h3 | H_h5 | H_l2 | H_l3 | H_l4 |
| --- | --- | --- | --- | --- | --- | --- | --- | --- | --- |
| p__Bacteria_unclassified | 404655.62 | 465131.61 | 461715.76 | 364600.72 | 427723.45 | 432655.16 | 447727.52 | 429182.02 | 422198.27 |
| p__Firmicutes | 163008.47 | 168292.99 | 168614.97 | 287390.47 | 238644.47 | 201868.90 | 255799.82 | 180232.77 | 204673.57 |
| p__Bacteroidetes | 125270.65 | 98523.59 | 93139.31 | 87984.91 | 76985.09 | 102507.37 | 57100.77 | 130779.86 | 103750.78 |
| p__Proteobacteria | 23629.75 | 18906.01 | 24709.77 | 13148.75 | 15310.49 | 20794.81 | 27548.88 | 26592.01 | 21602.35 |
| p__Fibrobacteres | 32893.92 | 39841.35 | 39830.81 | 21004.52 | 13231.92 | 1008.24 | 391.24 | 579.50 | 14501.80 |
| p__Bacteria_noname | 7551.06 | 5585.26 | 5284.14 | 9696.48 | 10623.73 | 6614.94 | 8739.99 | 7614.34 | 6326.70 |
| p__Spirochaetes | 12580.29 | 2449.43 | 4423.05 | 11577.33 | 5649.14 | 13527.26 | 1221.10 | 6908.44 | 6096.59 |
| p__Actinobacteria | 4806.96 | 2932.75 | 5250.36 | 3112.89 | 6796.89 | 3870.35 | 4159.32 | 4719.88 | 3914.40 |
| p__Tenericutes | 2305.74 | 2432.78 | 1572.27 | 3214.37 | 1139.57 | 3222.52 | 2551.45 | 2604.68 | 4983.32 |
| p__Lentisphaerae | 2530.35 | 2579.08 | 1798.68 | 2018.31 | 7517.97 | 2380.15 | 213.95 | 572.34 | 565.77 |
| p__Verrucomicrobia | 870.78 | 2457.88 | 1314.68 | 2436.06 | 3911.11 | 2573.94 | 1391.36 | 3812.80 | 1100.12 |
| p__Candidatus_Melainabacteria | 2334.58 | 1565.49 | 1862.36 | 2155.43 | 2593.35 | 3149.22 | 1938.15 | 351.11 | 2172.35 |
| p__Elusimicrobia | 1522.77 | 5070.33 | 2225.66 | 152.43 | 193.23 | 206.35 | 22.99 | 114.85 | 207.35 |
| p__Fusobacteria | 1007.16 | 764.35 | 580.01 | 583.19 | 645.49 | 852.40 | 638.88 | 424.84 | 731.47 |
| p__Chloroflexi | 465.66 | 388.77 | 373.95 | 691.04 | 602.92 | 496.44 | 494.89 | 340.08 | 407.01 |
| p__Chlamydiae | 295.08 | 352.14 | 354.83 | 427.32 | 489.13 | 491.44 | 394.18 | 394.14 | 424.03 |
| p__Planctomycetes | 353.51 | 348.11 | 405.81 | 408.76 | 722.79 | 440.85 | 197.10 | 267.56 | 252.98 |
| p__Candidatus_Saccharibacteria | 275.73 | 99.75 | 228.51 | 154.59 | 165.11 | 84.50 | 550.55 | 66.92 | 361.76 |
| p__Synergistetes | 155.32 | 174.71 | 158.93 | 357.85 | 236.80 | 200.79 | 303.38 | 144.26 | 181.52 |
| p__Cyanobacteria | 267.36 | 199.30 | 131.82 | 120.57 | 235.47 | 213.82 | 143.36 | 178.66 | 206.85 |
| Others | 1712.36 | 1462.99 | 1364.49 | 1494.29 | 2145.34 | 1818.56 | 1236.09 | 1387.70 | 1090.03 |

**Supplementary table 2:** the bacterial abundance at the phylum level in each group.

| Phylum | H_h | H_l | H_m |
| --- | --- | --- | --- |
| p__Bacteria_unclassified | 408326.44 | 433035.94 | 443834.33 |
| p__Firmicutes | 242634.61 | 213568.72 | 166638.81 |
| p__Bacteroidetes | 89159.12 | 97210.47 | 105644.52 |
| p__Proteobacteria | 16418.02 | 25247.75 | 22415.18 |
| p__Fibrobacteres | 11748.23 | 5157.51 | 37522.03 |
| p__Bacteria_noname | 8978.38 | 7560.34 | 6140.15 |
| p__Spirochaetes | 10251.24 | 4742.04 | 6484.26 |
| p__Actinobacteria | 4593.38 | 4264.53 | 4330.03 |
| p__Tenericutes | 2525.49 | 3379.82 | 2103.59 |
| p__Lentisphaerae | 3972.14 | 450.69 | 2302.70 |
| p__Verrucomicrobia | 2973.70 | 2101.43 | 1547.78 |
| p__Candidatus_Melainabacteria | 2632.66 | 1487.20 | 1920.81 |
| p__Elusimicrobia | 184.01 | 115.06 | 2939.59 |
| p__Fusobacteria | 693.69 | 598.40 | 783.84 |
| p__Chloroflexi | 596.80 | 413.99 | 409.46 |
| p__Chlamydiae | 469.30 | 404.12 | 334.02 |
| p__Planctomycetes | 524.13 | 239.21 | 369.15 |
| p__Candidatus_Saccharibacteria | 134.73 | 326.41 | 201.33 |
| p__Synergistetes | 265.15 | 209.72 | 162.99 |
| p__Cyanobacteria | 189.95 | 176.29 | 199.49 |
| Others | 1819.40 | 1237.94 | 1513.28 |

**Supplementary table 3:** the bacterial abundance at the genus level in each sample.

| Genus | H_m3 | H_m4 | H_m5 | H_h2 | H_h3 | H_h5 | H_l2 | H_l3 | H_l4 |
| --- | --- | --- | --- | --- | --- | --- | --- | --- | --- |
| g__Bacteria_unclassified | 143090.18 | 184960.95 | 194687.89 | 126405.63 | 157095.40 | 165318.65 | 184375.36 | 152751.60 | 153927.85 |
| g__Clostridium | 50330.96 | 51352.38 | 52127.18 | 71971.63 | 59776.77 | 66035.53 | 69514.34 | 57633.44 | 65409.00 |
| g__Bacteroides | 58892.84 | 52728.47 | 67826.18 | 50877.78 | 46238.65 | 69369.89 | 29707.81 | 89295.73 | 44239.89 |
| g__Prevotella | 88044.38 | 51147.11 | 32053.78 | 24744.05 | 18694.04 | 23199.24 | 29698.64 | 26350.39 | 57995.29 |
| g__Firmicutes_noname | 28789.33 | 33471.54 | 30845.15 | 48062.10 | 39188.36 | 37486.68 | 38505.71 | 33854.77 | 34085.62 |
| g__Ruminococcus | 28607.43 | 20902.92 | 24545.44 | 44547.46 | 56752.72 | 29283.78 | 27350.77 | 25104.14 | 26629.16 |
| g__Clostridiales_noname | 21443.37 | 24620.34 | 20716.44 | 32827.85 | 36593.91 | 24371.86 | 39622.94 | 24058.78 | 23955.84 |
| g__Fibrobacter | 39832.42 | 52999.22 | 52081.27 | 25689.27 | 16652.73 | 1470.36 | 624.22 | 874.23 | 18629.48 |
| g__Alistipes | 11671.43 | 21919.24 | 22715.31 | 12416.55 | 13223.28 | 28532.64 | 7067.42 | 36162.18 | 11828.56 |
| g__Ruminococcaceae_noname | 10934.10 | 12858.39 | 11880.00 | 36036.04 | 23222.79 | 18882.58 | 16943.63 | 10675.42 | 18378.63 |
| g__Lachnospiraceae_noname | 13895.67 | 14373.32 | 13116.49 | 17715.47 | 19264.79 | 13761.89 | 22579.31 | 16011.84 | 15418.45 |
| g__Bacteria_noname | 13643.80 | 11234.73 | 10456.79 | 15213.77 | 17845.06 | 12067.03 | 16509.71 | 15353.23 | 11959.81 |
| g__Eubacterium | 9119.07 | 9389.94 | 9067.65 | 13284.48 | 11323.98 | 16781.85 | 11821.35 | 8785.84 | 9665.11 |
| g__Treponema | 17472.71 | 3608.53 | 9828.77 | 15167.64 | 8250.63 | 15641.16 | 953.03 | 9893.31 | 15714.61 |
| g__Oscillibacter | 6572.05 | 9167.75 | 10807.64 | 9887.62 | 9295.35 | 8872.99 | 16699.14 | 7014.51 | 11837.64 |
| g__Roseburia | 7230.48 | 8683.47 | 7803.26 | 9047.96 | 9537.96 | 7987.43 | 17462.93 | 10295.51 | 10012.28 |
| g__Faecalibacterium | 5711.60 | 6848.33 | 5669.65 | 12833.32 | 13140.64 | 7048.25 | 15322.11 | 4729.28 | 7014.24 |
| g__Lachnoclostridium | 6841.52 | 8202.48 | 8062.35 | 9291.28 | 9954.38 | 7501.08 | 12086.61 | 8138.59 | 7598.10 |
| g__Clostridiales_unclassified | 5938.16 | 7379.69 | 7113.28 | 7174.58 | 8206.56 | 6945.78 | 13732.79 | 8298.26 | 8328.10 |
| g__Blautia | 5819.13 | 7066.65 | 7287.07 | 8336.87 | 7610.83 | 7032.62 | 11727.28 | 7633.60 | 7792.53 |
| Others | 214612.49 | 226643.23 | 216648.58 | 221198.92 | 233694.62 | 231386.70 | 230459.86 | 244354.12 | 235328.83 |

**Supplementary table 4:** the bacterial abundance at the genus level in each group.

| Genus | H_h | H_l | H_m |
| --- | --- | --- | --- |
| g__Bacteria_unclassified | 149606.56 | 163684.93 | 174246.34 |
| g__Clostridium | 65927.98 | 64185.59 | 51270.17 |
| g__Bacteroides | 55495.44 | 54414.48 | 59815.83 |
| g__Prevotella | 22212.44 | 38014.77 | 57081.76 |
| g__Firmicutes_noname | 41579.05 | 35482.03 | 31035.34 |
| g__Ruminococcus | 43527.99 | 26361.35 | 24685.26 |
| g__Clostridiales_noname | 31264.54 | 29212.52 | 22260.05 |
| g__Fibrobacter | 14604.12 | 6709.31 | 48304.30 |
| g__Alistipes | 18057.49 | 18352.72 | 18768.66 |
| g__Ruminococcaceae_noname | 26047.14 | 15332.56 | 11890.83 |
| g__Lachnospiraceae_noname | 16914.05 | 18003.20 | 13795.16 |
| g__Bacteria_noname | 15041.96 | 14607.58 | 11778.44 |
| g__Eubacterium | 13796.77 | 10090.77 | 9192.22 |
| g__Treponema | 13019.81 | 8853.65 | 10303.34 |
| g__Oscillibacter | 9351.99 | 11850.43 | 8849.15 |
| g__Roseburia | 8857.78 | 12590.24 | 7905.73 |
| g__Faecalibacterium | 11007.40 | 9021.88 | 6076.53 |
| g__Lachnoclostridium | 8915.58 | 9274.44 | 7702.12 |
| g__Clostridiales_unclassified | 7442.30 | 10119.72 | 6810.38 |
| g__Blautia | 7660.11 | 9051.14 | 6724.28 |
| Others | 228760.08 | 236714.27 | 219301.43 |
